# Increased CD112 Expression in Methylcholanthrene-Induced Tumors in CD155-Deficient Mice

**DOI:** 10.1371/journal.pone.0112415

**Published:** 2014-11-10

**Authors:** Yoko Nagumo, Akiko Iguchi-Manaka, Yumi Yamashita-Kanemaru, Fumie Abe, Günter Bernhardt, Akira Shibuya, Kazuko Shibuya

**Affiliations:** 1 Faculty of Life and Environmental Sciences, University of Tsukuba, Tsukuba, Japan; 2 Department of Immunology, Faculty of Medicine, University of Tsukuba, Tsukuba, Japan; 3 Department of Breast and Endocrine Surgery, Faculty of Medicine, University of Tsukuba, Tsukuba, Japan; 4 Japan Science and Technology Agency, Core Research for Evolutional Science and Technology (CREST), University of Tsukuba, Tsukuba, Japan; 5 Life Science Center of Tsukuba Advanced Research Alliance (TARA), University of Tsukuba, Tsukuba, Japan; 6 Institute of Immunology, Hannover Medical School, Hannover, Germany; Ohio State University, United States of America

## Abstract

Tumor recognition by immune effector cells is mediated by antigen receptors and a variety of adhesion and costimulatory molecules. The evidence accumulated since the identification of CD155 and CD112 as ligands for DNAM-1 in humans and mice has suggested that the interactions between DNAM-1 and its ligands play an important role in T cell– and natural killer (NK) cell–mediated recognition and lysis of tumor cells. We have previously demonstrated that methylcholanthrane (MCA) accelerates tumor development in DNAM-1–deficient mice, and the *Cd155* level on MCA-induced tumors is significantly higher in DNAM-1–deficient mice than in wild-type (WT) mice. By contrast, *Cd112* expression on the tumors is similar in WT and DNAM-1-deficient mice, suggesting that CD155 plays a major role as a DNAM-1 ligand in activation of T cells and NK cells for tumor immune surveillance. To address this hypothesis, we examined MCA-induced tumor development in CD155-deficient mice. Unexpectedly, we observed no significant difference in tumor development between WT and CD155-deficient mice. Instead, we found that *Cd112* expression was significantly higher in the MCA-induced tumors of CD155-deficient mice than in those of WT mice. We also observed higher expression of DNAM-1 and lower expression of an inhibitory receptor, TIGIT, on CD8^+^ T cells in CD155-deficient mice. These results suggest that modulation of the expression of receptors and CD112 compensates for CD155 deficiency in immune surveillance against MCA-induced tumors.

## Introduction

Cancer immune surveillance to suppress tumor development is an important host protection process. Several immune effector cell types and secreted cytokines play a critical role in this process. Among them, cytotoxic T lymphocytes (CTL) and natural killer (NK) cells are major players in cell-mediated immunity against tumors [1], [2]. Interaction of cell surface receptors on CTL and NK cells with their respective ligands expressed on tumors activates the CTL and NK cells [2], [3], resulting in their secretion of cytokines and cytotoxicity against tumors [4], [5].

The leukocyte adhesion molecule DNAX accessory molecule-1 (DNAM-1, also known as CD226) is a member of the immunoglobulin (Ig) superfamily and is constitutively expressed on most CD4^+^ and CD8^+^ T cells, NK cells, monocytes, and macrophages [6]. The poliovirus receptor (PVR) CD155 and another member of the same family, CD112 (PVR-related family 2 [PRR-2], also called nectin-2), are the ligands of DNAM-1 in humans and mice [7]–[9]. Interactions between DNAM-1 on NK cells and CD8^+^ T cells and CD112 and CD155 on tumor cells augment cell-mediated cytotoxicity and cytokine production [7], [8].

CD155 and CD112 are present on various types of epithelial and endothelial cells in many tissues [10], [11]. A number of studies have demonstrated that CD155 and CD112 are overexpressed on certain hematopoietic and nonhematopoietic tumors [12]–[17], suggesting that DNAM-1 ligand expression might be induced by tumorigenesis and might stimulate CTL- and NK cell–mediated tumor immunity. Of note, CD155 and CD112 also bind TIGIT (T cell immunoreceptor with Ig and ITIM [immunoreceptor tyrosine-based inhibitory motif] domains), which is expressed on T cells and NK cells and mediates an inhibitory signal (either directly or indirectly) in these cells [18], [19]. CD155 also binds an immunoreceptor, CD96 (also called T cell-activated increased late expression [TACTILE]), that is expressed on both activated T cells and NK cells [20]–[22]. Taken together, the available data suggest that CD155 might be a double-edged sword balancing tumor growth and elimination.

We have previously demonstrated that the chemical carcinogens methylcholanthrane (MCA) and 7,12-dimethylbenz[a]anthracene (DMBA) result in significantly greater development of fibrosarcoma and papilloma, respectively, in DNAM-1–deficient mice than in wild-type (WT) mice [23]. Interestingly, we found that although *Cd155* expression on MCA-induced fibrosarcomas was significantly higher in DNAM-1–deficient mice than in WT mice, *Cd112* expression was similar, suggesting that CD155, rather than CD112, is the tumor ligand involved in DNAM-1–mediated immune surveillance against MCA-induced fibrosarcoma. In the present study, we used CD155-deficient mice to examine the role of CD155 on MCA-induced fibrosarcomas in tumor immune surveillance.

## Materials and Methods

### Mice

C57BL/6N and BALB/c mice were purchased from CLEA (Tokyo, Japan). CD155-deficient C57BL/6N and BALB/c mice were described previously [24]; they were additionally backcrossed twice with C57BL/6N and BALB/c mice, respectively (a total of 12 generations). All mice were housed and bred under specific-pathogen-free conditions at the Animal Resource Center of the University of Tsukuba. Animal experiments were carried out in a humane manner after receiving approval from the Animal Experiment Committee of the University of Tsukuba (Approval No.: 10-237, 11-231, 12-231), and in accordance with Fundamental Guideline for Proper Conduct of Animal Experiment and Related Activities in Academic Research Institutions under the Jurisdiction of the Ministry of Education, Culture, Sports, Science and Technology, and Japanese Act on Welfare and Management of Animals (No.105).

### Flow cytometry

CD4^+^ T, CD8^+^ T, and NK cells from *peripheral* blood were analyzed using an LSRFortessa flow cytometer (BD Biosciences, San Jose, CA). The anti-DNAM-1 monoclonal antibody (mAb) TX42 (rat IgG2a) was generated in our laboratory [13]. All other antibodies for flow cytometry analyses were purchased from BD Biosciences. CD8^+^ T cells were purified by magnetic separation from spleen cells on a Mini-MACS system (Miltenyi Biotec, Bergisch Gladbach, Germany). Purified CD8^+^ T cells were cultured in the presence of 20 ng/ml exogenous IL-2 (BD Biosciences) in a 24-well plate coated with 1 µg/ml of anti-CD3e mAb (BD Biosciences). The cells were harvested at various time points, and the cell surface molecules were analyzed by flow cytometry. Antibodies against TIGIT and CD96 were from eBioscience (San Diego, CA) and R&D Systems (Minneapolis, MN), respectively. Cell staining and flow cytometry were performed according to standard procedures. The CellQuest (BD Biosciences) and FlowJo (Tree Star, Inc., Ashland, OR) programs were used for data acquisition and analysis.

### Tumor growth assay and survival of mice

Groups of 10–20 WT or CD155-deficient male mice (8–12 week-old) were injected subcutaneously (s.c.) in the back with 5 or 100 µg (as specified in the figure legends) MCA (Sigma-Aldrich, St. Louis, MO) dissolved in 0.1 ml corn oil (Sigma-Aldrich) after anesthetization (7∶3 mixture of polyethylene glycol and isoflurane). Mice were examined at least once a week for tumor size with a caliper square as previously described [13]. Mouse survival was closely monitored 3 times per week during the experimental period and the humane endpoint was applied for euthanization by excess anesthetization.

### Quantitative real-time PCR (qRT-PCR)

Total RNA was extracted from fibrosarcomas and naive tissues with the Isogen reagent (Nippon Gene, Tokyo, Japan). For reverse transcription, we used 2 µg of total RNA and a High-Capacity cDNA Reverse Transcription kit (Applied Biosystems, Carlsbad, CA) in a final volume of 20 µl. qRT-PCR was performed on a 7500 FAST Real-Time PCR System (Applied Biosystems) with Platinum SYBR Green qPCR SuperMix-UDG (Invitrogen, Grand Island, NY). The primers were as follows: *Ifng*, 5′-TCAAGTGGCATAGATGTGGAAGAA-3′ (forward) and 5′-TGGCTCTGCAGGATTTTCATG-3′ (reverse); *Il1b*, 5′-TGAAGCAGCTATGGCAACTG-3′ (forward) and 5′-CAGGTCAAAGGTTTGGAAGC-3′ (reverse); *Il12b*, 5′-GGAGACCCTGCCCATTGAACT-3′ (forward) and 5′-CAACGTTGCATCCTAGGATCG-3 (reverse); *Il10*, 5′-GCTGGACAACATACTGCTAACC-3′ (forward) and 5′-ATTTCCGATAAGGCTTGGCAA-3′ (reverse); *Cd112d*, 5′-CTCTGTGGATCGAATGGTCA-3′ (forward) and 5′-GGCAGCGATAATACCTCCAA-3′ (reverse); *Cd226*, 5′-TCGCTCAGAGGCCATTACAG-3′ (forward) and 5′-CCCTGGGCTCTTTAAGTGGAA-3′ (reverse); *Tigit*, 5′-CTGATACAGGCTGCCTTCCT-3′ (forward) and 5′-TGGGTCACTTCAGCTGTGTC-3′ (reverse); *Cd96*, 5′-TCCCCAATATGGCCTCTACTG-3′ (forward) and 5′-GACTGTAGTCTTGATGCCTTCTG-3 (reverse). The level of the *β-actin* or *Cd2* transcripts were measured as internal controls. *β-actin,* 5′-GGCTGTATTCCCCTCCATCG-3′ (forward) and 5′-CCAGTTGGTAACAATGCCATGT-3′ (reverse); *Cd2,* 5′- TGGTAACTCATGTTCTTCTGG-3′ (forward) and 5′- GTAATGGTGTATGGCACAAATG-3′ (reverse). PCR conditions were as follows: an initial denaturation step at 95°C for 10 min, followed by 40 cycles at 95°C for 15 s and 60°C for 1 min. Data were analyzed by the 2^−ΔΔCt^ method. All values were determined in triplicate.

### Statistics

The survival of mice was analyzed by the Kaplan–Meier survival method followed by the log-rank test. All other statistical analyses were performed using the unpaired Student’s *t* test. *P*<0.05 was considered statistically significant.

## Results

### MCA-induced tumor development is similar in WT and CD155-deficient mice

**Figure 1:**
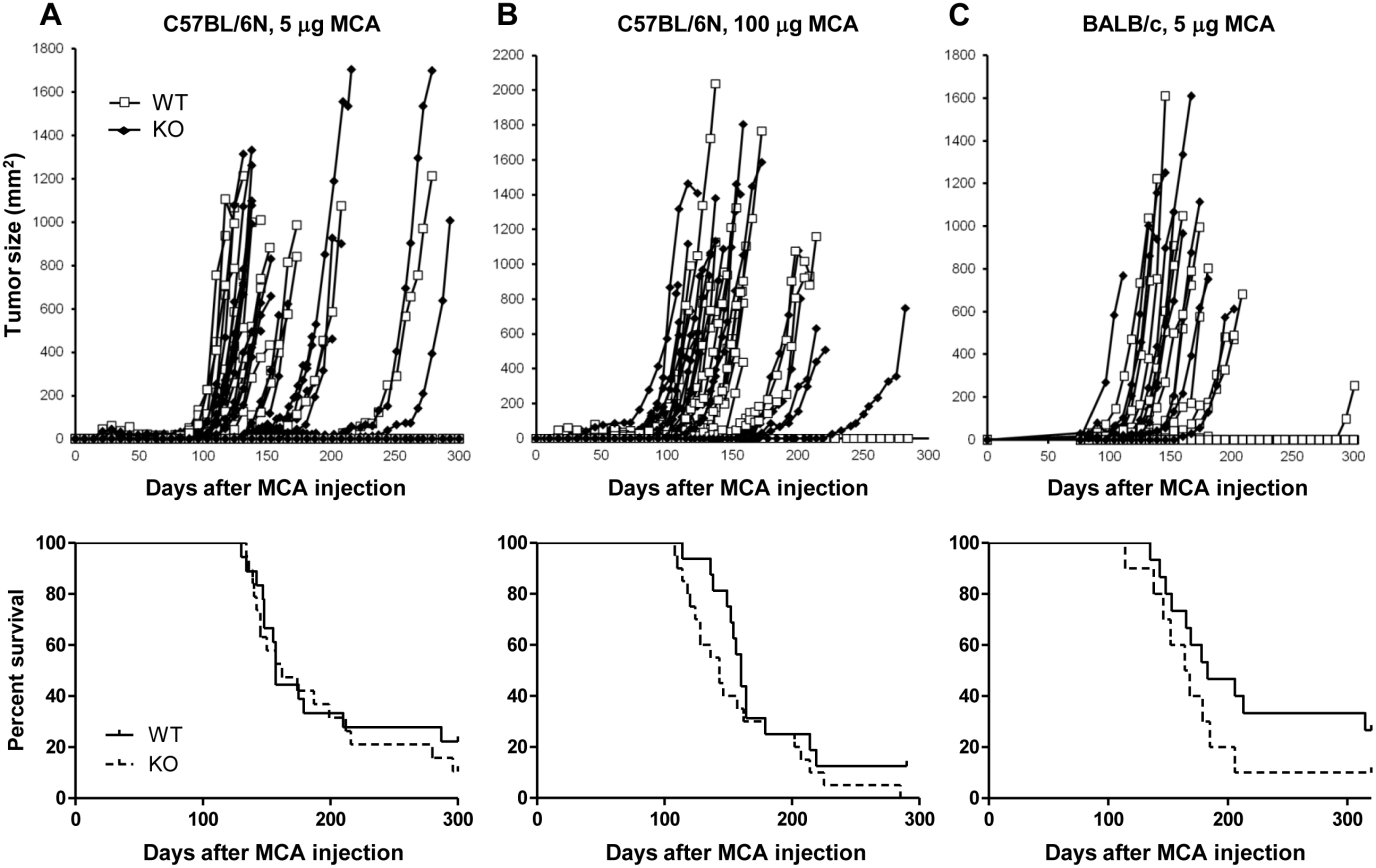
MCA-induced tumor development in WT and CD155-deficient mice. (A, B) WT (*n* = 18 and 16, respectively) or CD155-deficient (KO; *n* = 19 and 20, respectively) C57BL/6N mice were injected s.c. with 5 µg (A) or 100 µg (B) methylcholanthrane (MCA) on day 0. (C) WT (*n* = 15) or KO (*n* = 10) BALB/c mice were injected s.c. with 5 µg MCA on day 0. Tumor size in each mouse was measured once a week. Tumor size (top) and survival data (bottom) are shown.

To examine the effect of CD155 on MCA-induced fibrosarcoma in tumor immune surveillance, WT and CD155-deficient mice in the C57BL/6N background were injected with MCA. Unexpectedly, both groups showed a similar course of fibrosarcoma development 80–250 days after MCA injection, and their survival rates were similar (**Fig. 1A, B**). Similar results were obtained for WT and CD155-deficient mice in the BALB/c background (**Fig. 1C**). These results suggest that, although DNAM-1 expressed on T cells and NK cells plays an important role in immune surveillance against MCA-induced fibrosarcoma, the counterpart of DNAM-1 on the fibrosarcoma may not be CD155 alone.

### Expression of cytokine genes is similar in tumors from WT and CD155-deficient mice

**Figure 2:**
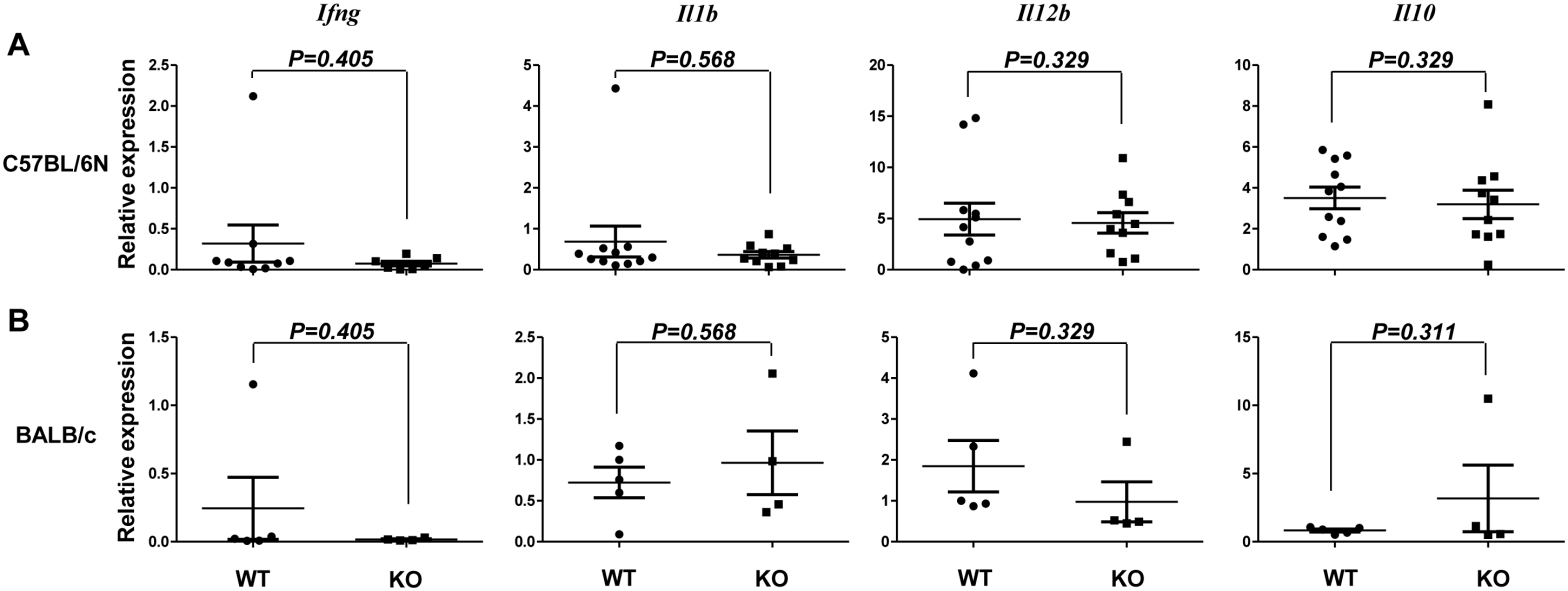
Relative cytokine mRNA levels in MCA-induced tumors. (A, B) Fibrosarcomas induced by 5 µg MCA in WT or CD155-deficient (KO) C57BL/6N (A) or BALB/c (B) mice were resected from each mouse and subjected to quantitative qRT-PCR for transcripts of the indicated cytokines. Horizontal bars represent means and error bars represent means ± SEM. *P* Values for Student’s *t* test are shown.

Cytokines expressed in immune cells infiltrating tumor tissues are involved in tumor immune responses. Because there was no difference in MCA-induced tumor development between WT and CD155-deficient mice, we next investigated cytokine expression in tumor tissues from the two types of mice. qRT-PCR revealed that the levels of mRNA for the proinflammatory cytokines *Ifng, Il1b,* and *Il12b* and the anti-inflammatory cytokine *Il10* in fibrosarcomas induced by 5 µg MCA were similar in WT and CD155-deficient mice in both C57BL/6N and BALB/c backgrounds (**Fig. 2**). These results suggest that cytokine expression levels in immune cells infiltrating into tumors were similar in CD155-defficient mice and WT mice.

### *Cd112* expression is increased in MCA-induced fibrosarcoma in CD155-deficient mice

**Figure 3:**
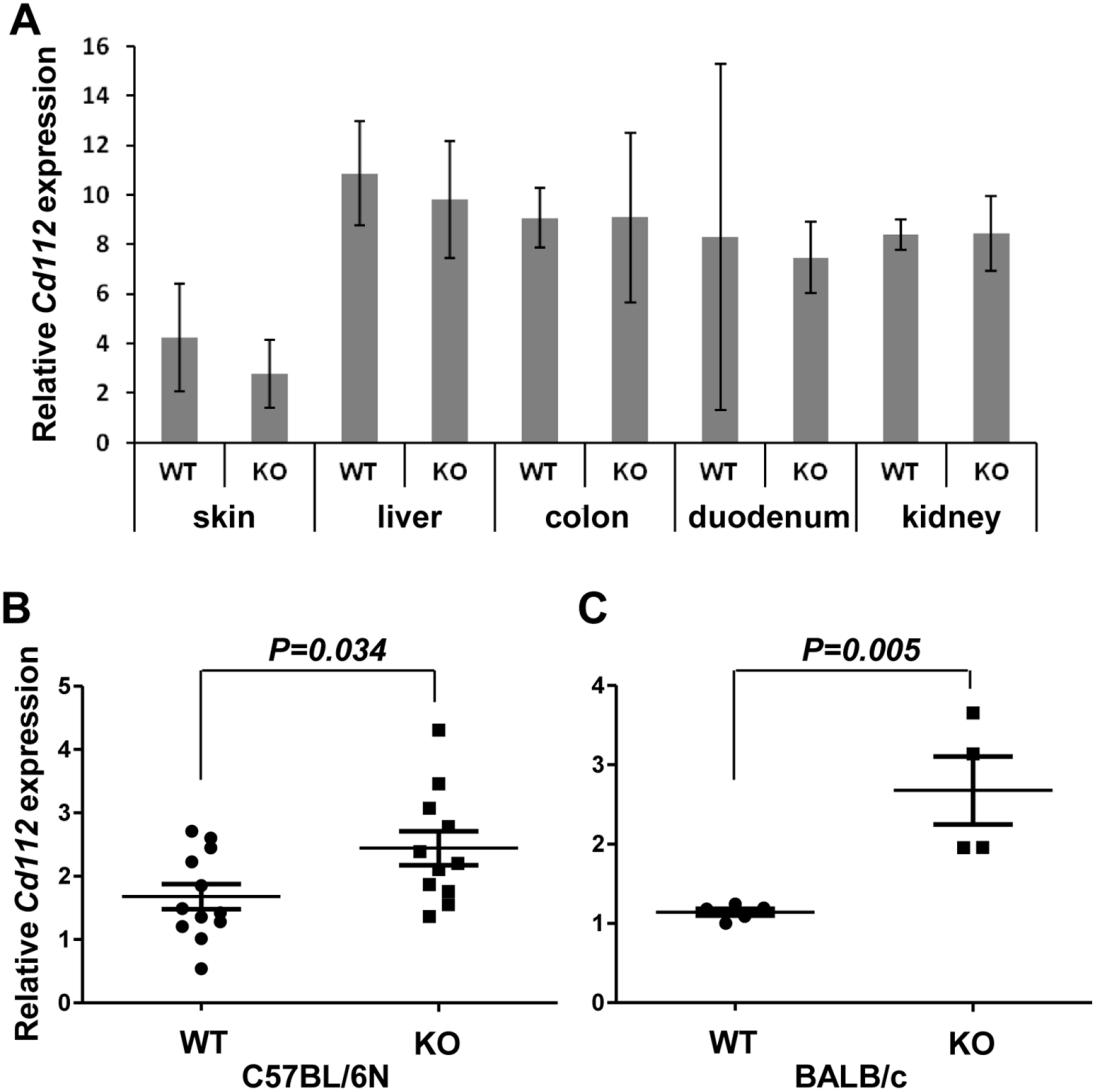
Relative *Cd112* mRNA levels in organs and MCA-induced tumors. (A) Tissues from WT (*n* = 3) or CD155-deficient (KO; *n* = 3) mice were subjected to quantitative qRT-PCR for *Cd112*. Error bars represent means ± SD. (B, C) Fibrosarcomas induced in WT or CD155-deficient C57BL/6N (B) or BALB/c (C) mice by 5 µg MCA were resected from each mouse and subjected to qRT-PCR for *Cd112*. Horizontal bars represent means and error bars represent means ± SEM. *P* Values for Student’s *t* test are shown.

We next examined the expression of *Cd112* in MCA-induced fibrosarcoma in WT and CD155-defficient mice. In naïve C57BL/6N mice, *Cd112* expression was similar in several organs, including the skin, in WT and CD155-deficient mice (**Fig. 3A**). However, in MCA-induced fibrosarcoma, *Cd112* expression was significantly higher in CD155-deficient mice than in WT mice (**Fig. 3B**). Similar results were obtained for CD155-deficient mice in the BALB/c background (**Fig. 3C**). These results suggest that *Cd112* expression is upregulated in MCA-induced fibrosarcoma in CD155-deficient mice during the transformation.

### DNAM-1 and CD96 expressions on resting T cells are increased in CD155-deficient mice

**Figure 4:**
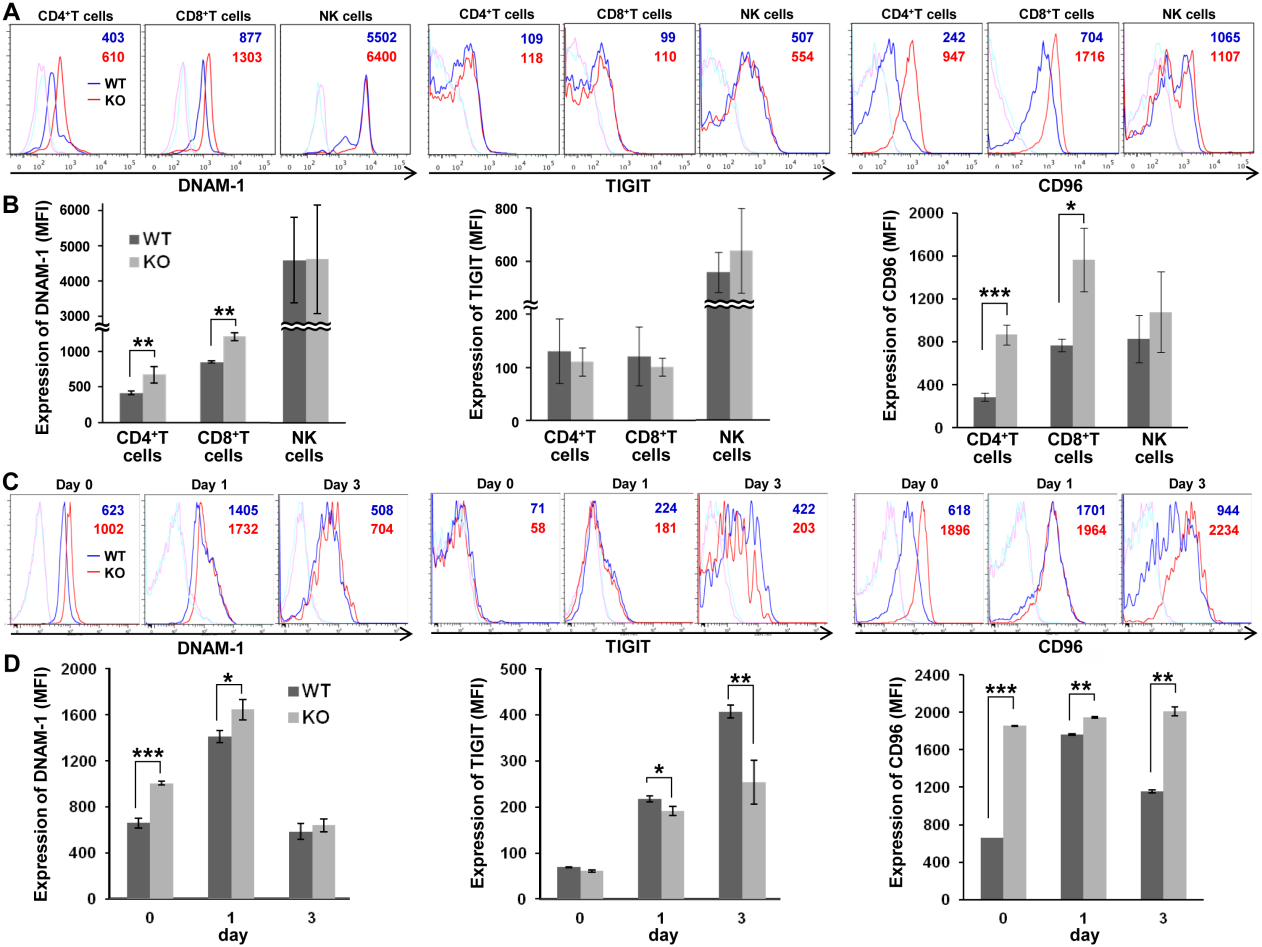
Expression of CD155 ligands on resting and activated T cells from WT and CD155-deficient mice. (A) Peripheral blood lymphocytes from WT (*n* = 3) or CD155-deficient (KO; *n* = 3) C57BL/6N mice were stained with anti-DNAM-1, anti-TIGIT, or anti-CD96 antibodies (WT; blue lines, KO; red lines) or control antibodies (WT; light blue lines, KO; pink lines), and analyzed by flow cytometry. The numbers (WT; in blue, KO; in red) indicate the mean fluorescence intensity (MFI) of DNAM-1, TIGIT, and CD96 staining. Representative data are shown. (B) MFI was used to analyze DNAM-1 expression on CD4^+^ T, CD8^+^ T, and NK cells as in (A). Error bars represent means ± SD. (C) CD8^+^ T cells purified from spleen were activated with anti-CD3 antibody and IL-2 for the indicated number of days. Cells were stained and analyzed by flow cytometry, as described in (A). (D) The expression of DNAM-1, TIGIT, and CD96 on CD8^+^ T cells is shown as MFI as in (C). *, *P*<0.05; **, *P*<0.01; ***, *P*<0.001.

A previous report demonstrated that DNAM-1 expression on T cells was significantly higher in CD155-deficient mice than in WT mice [25]. Consistent with this, our flow cytometry analysis demonstrated significantly higher expression of DNAM-1 on peripheral blood CD4^+^ and CD8^+^ T cells **(** **Fig. 4A**). Fluorescence intensity analysis confirmed a significant increase in DNAM-1 expression on CD4^+^ and CD8^+^ T cells (**Fig. 4B**). At the same time, we also analyzed the expressions of TIGIT and CD96 on those cells and found that CD96 but not TIGIT expression on CD4^+^ and CD8^+^ T cells was also significantly higher in CD155-deficient mice than in WT mice. As to NK cells, however, we did not observe a difference of these molecules’ expression between in WT and in CD155-deficient mice. TIGIT was markedly expressed on NK cells compared to CD4^+^ and CD8^+^ T cells, although there was no significant difference between WT and CD155-deficient mice.

### Receptor modulation by CD8^+^ T cell activation

As CD155 binds CD96, TIGIT, and DNAM-1, we examined the expression of these receptors on activated CD8^+^ T cells. CD8^+^ T cells from the spleens of WT and CD155-deficient mice were stimulated with anti-CD3 mAb in the presence of IL-2. DNAM-1 and CD96 expression was significantly higher on resting CD155-deficient CD8^+^ T cells than on resting WT CD8^+^ T cells, whereas TIGIT expression was not detected (**Fig. 4C, D**). Activated CD8^+^ T cells increased expression of TIGIT on both WT and CD155-deficient CD8^+^ T cells after stimulation. Upon stimulation, the levels of DNAM-1 and CD96 were still significantly higher on CD155-deficient than on WT CD8^+^ T cells, whereas TIGIT expression was significantly lower on CD155-deficient CD8^+^ T cells than on WT CD8^+^ T cells (**Fig. 4C, D**).

To analyze the expressions of DNAM-1, TIGIT, and CD96 on tumor-resident immune cells, fibrosarcomas induced by 5 µg MCA in WT or CD155-deficient mice were resected and subjected to quantitative RT-PCR by using *Cd2* expression, which is specifically expressed on T cells and NK cells, but not on non-hematopoietic cells, for normalization. The average tumor size and time points of resection after MCA injection were comparable between two groups. However, we did not observe different expression levels of those receptors in tumors between WT and CD155-deficient mice (**Fig. S1**).

## Discussion

CD155 and CD112 are involved in tumor immune surveillance, because ectopic expression of CD155 or CD112 on tumors can induce cell-mediated cytotoxicity [13]. Although the binding affinities of DNAM-1 to CD155 and CD112 are similar [8], CD155 rather than CD112 appears to play a predominant role in DNAM-1-dependent NK cell triggering [7]. CD112, but not CD155, mediates not only heterophilic but also homophilic binding. This ability may adversely affect DNAM-1 binding to CD112, suggesting that DNAM-1 may prefer CD155 to CD112 as a physiological ligand [8], [26].

**Figure 5:**
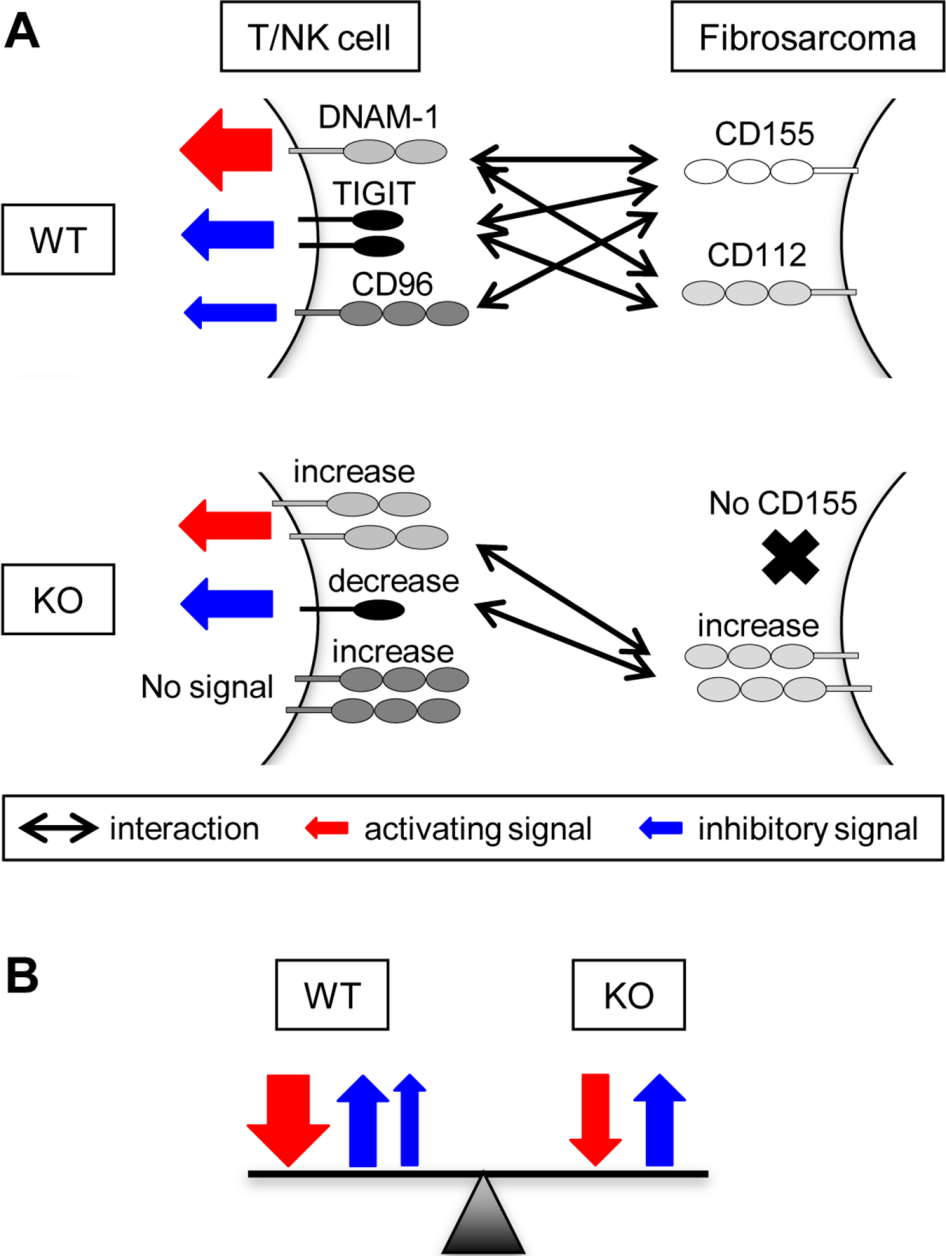
A hypothetical model for interactions between activating/inhibitory receptors DNAM-1, TIGIT and CD96 on T or NK cells and CD155/CD112 ligands expressed on MCA-induced fibrosarcoma in WT or CD155-deficient mice. (A) DNAM-1 and TIGIT bind to both of CD155 and CD112, while CD96 interacts with CD155 only. Each receptor-ligand interaction transduces either activating or inhibitory signal, as shown by the red or blue arrow, respectively. The modulation of the receptors and ligand expression on CD155-deficient (KO) fibrosarcoma are indicated. (B) The sums of the activating and inhibitory signals are similar between WT and KO.

Our previous study demonstrated that the development of MCA-induced fibrosarcoma and DMBA-induced papilloma was enhanced in DNAM-1-deficient mice, suggesting that DNAM-1 on T cells, NK cells, or both plays an important role in immune surveillance against DNAM-1 ligand–expressing tumors [23]. In this study, we used CD155-deficient mice to examine whether CD155 on MCA-induced fibrosarcoma plays a reciprocal role in tumor immunity. Unexpectedly, however, we found that MCA-induced tumors developed similarly in WT and CD155-deficient mice. We suggest several possible explanations of why the absence of CD155 on tumors did not affect tumor immunity (**Fig. 5**). First, *Cd112* expression in fibrosarcomas was significantly higher in CD155-deficient mice than in WT mice, suggesting that DNAM-1 interaction with CD112 compensated for the loss of the DNAM-1–CD155 interaction. CREB and c-Jun may regulate *Cd112* transcription [27] and are often activated in cancers [28], [29]. The relative amounts of *Cd112* mRNA are higher in poorly differentiated gastric cancer than in normal gastric tissue [13]. Second, DNAM-1 expression was significantly upregulated on CD4^+^ and CD8^+^ T cells from CD155-deficient mice, consistent with a previous report [25]. DNAM-1 upregulation in CD8^+^ T cells was independent of antigen-driven activation because it was already observed in resting T cells, but it was still upregulated after stimulation. We also found that, although TIGIT expression was upregulated after stimulation with anti-CD3 mAb in both WT and CD155-deficient CD8^+^ T cells, it was significantly lower in CD155-deficient cells than in WT cells. TIGIT is an inhibitory receptor that binds to both CD155 and CD112 [18]. Thus, upregulated expression of the activating receptor DNAM-1 and downregulated expression of the inhibitory receptor TIGIT should be favorable for cytotoxicity mediated by T cells, NK-cells, or both against tumors with upregulated CD112, even if the affinity of CD112 for binding DNAM-1 or TIGIT is lower than that of CD155. CD96 expression was also significantly higher on CD155-deficient CD8^+^ T cells than WT cells. Recent evidence indicates that CD96, which binds CD155 but not CD112, negatively regulates tumor immunity and cytokine secretion by NK cells [21]. Thus, the absence of CD155 on tumors would downregulate CD96-mediated NK cell inhibition, resulting in an increase in cytotoxicity against CD155-deficient tumors. It remains unclear how the expression of DNAM-1, TIGIT, CD96, and CD112 is modulated in CD155-deficient mice. A better understanding of the mechanisms that regulate the levels of these molecules may provide new insights into possible ways to improve cancer immunotherapy.

In contrast to the peripheral blood lymphocytes, we did not observe any different expression levels of DNAM-1, TIGIT, and CD96 in tumors after normalization by *Cd2* expression between WT and CD155-deficient mice. It is unclear at present how these results can be explained. However, it is possible that, during tumor development for more than 100 days, immune cell activation might be modified. For example, myeloid-derived suppressor cells (MDSC) in tumors tissues suppress both innate and adaptive immunities by secretion of various cytokines [30], which might affect the expression of the cell surface molecules.

We have previously shown that abrogating DNAM-1 activity on CD8+ T cells results in development of milder graft-versus-host disease (GVHD) [31], [32]. On the other hand, Seth et al. reported that the absence of CD155 aggravated acute GVHD, which is mainly caused by CD4^+^ T cells [33]. Although there are differences in the experimental settings between the two GVHD models, these observations and the results of our current study indicate that phenotypes of CD155 and DNAM-1 deficiencies are not two sides of the same coin.

## Supporting Information

Figure S1 Relative DNAM-1, TIGIT, and CD96 mRNA levels in MCA-induced tumors. Fibrosarcomas induced by 5 µg MCA in WT or CD155-deficient (KO) C57BL/6N mice were resected and subjected to quantitative RT-PCR for the expression of transcripts of the indicated receptors as described in Materials and Methods. (A) Tumor size, days of resection after MCA injection, and relative expressions of the transcripts are shown. (B) Relative expressions of indicated receptors are shown. Horizontal bars represent means and error bars represent means ± SEM. *P* Values for Student’s *t* test are shown.
(PPT)

Material S1 The ARRIVE Guidelines Checklist. We followed the ARRIVE (Animal Research: Reporting of *In Vivo* Experiments) guidelines and the ARRIVE Checklist is available as supporting information.
(PDF)
