# Supplementary figures and images for: Increased CD112 Expression in Methylcholanthrene-Induced Tumors in CD155-Deficient Mice
Source: PLoS One. 2014 Nov 10;9(11):e112415. doi: 10.1371/journal.pone.0112415 (PMC4226556; doi:10.1371/journal.pone.0112415)

## Slide 1
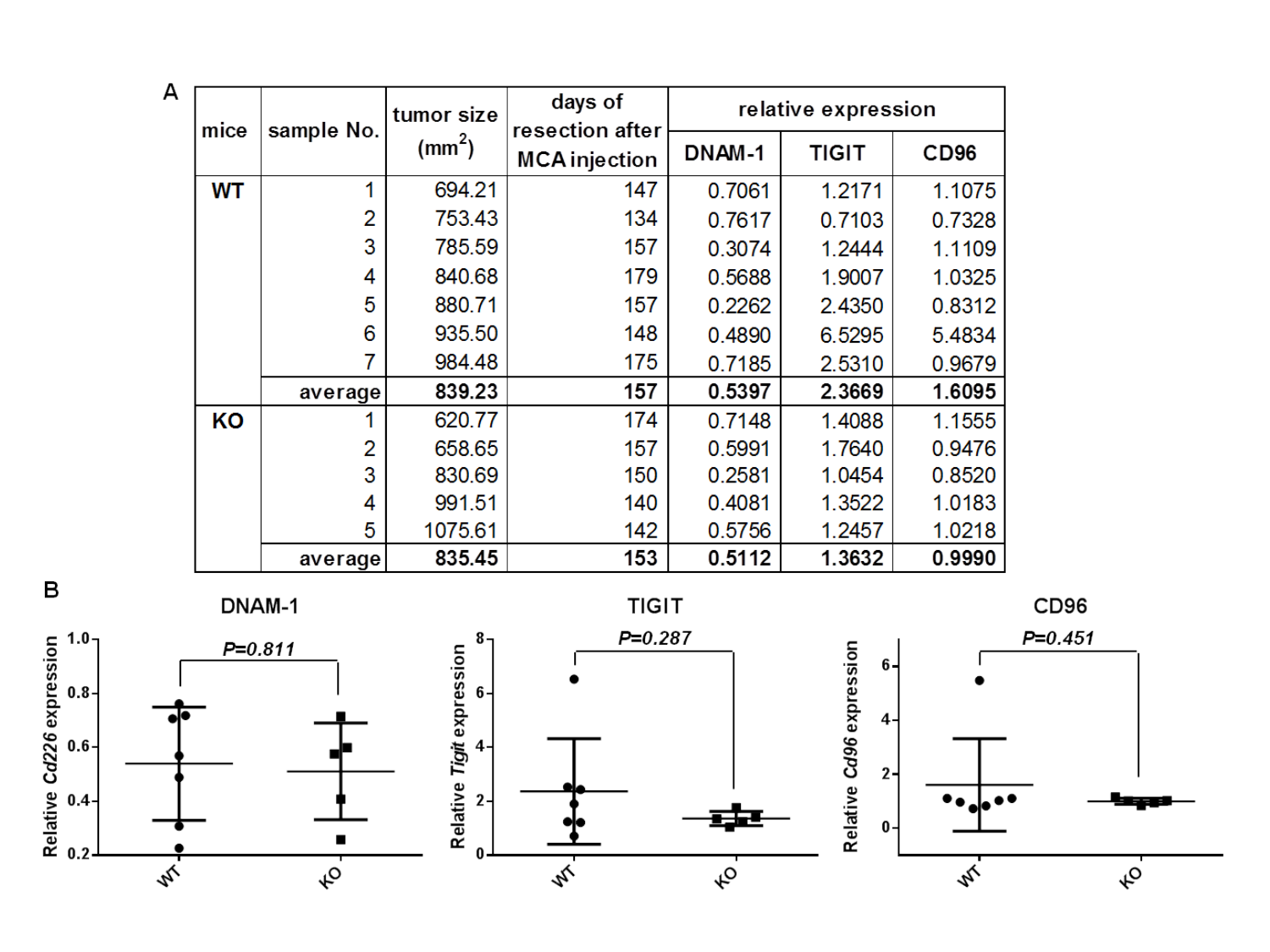

Supplement: Figure S1 — Relative DNAM-1, TIGIT, and CD96 mRNA levels in MCA-induced tumors. Fibrosarcomas induced by 5 µg MCA in WT or CD155-deficient (KO) C57BL/6N mice were resected and subjected to quantitative RT-PCR for the expression of transcripts of the indicated receptors as described in Materials and Methods. (A) Tumor size, days of resection after MCA injection, and relative expressions of the transcripts are shown. (B) Relative expressions of indicated receptors are shown. Horizontal bars represent means and error bars represent means ± SEM. P Values for Student’s t test are shown. (PPT) [file pone.0112415.s001.ppt]
